# Supplementary figures and images for: Integrated Transcriptome and Molecular Docking to Identify the Hub Superimposed Attenuation Targets of Curcumin in Breast Cancer Cells
Source: Int J Mol Sci. 2023 Aug 5;24(15):12479. doi: 10.3390/ijms241512479 (PMC10419115; doi:10.3390/ijms241512479)

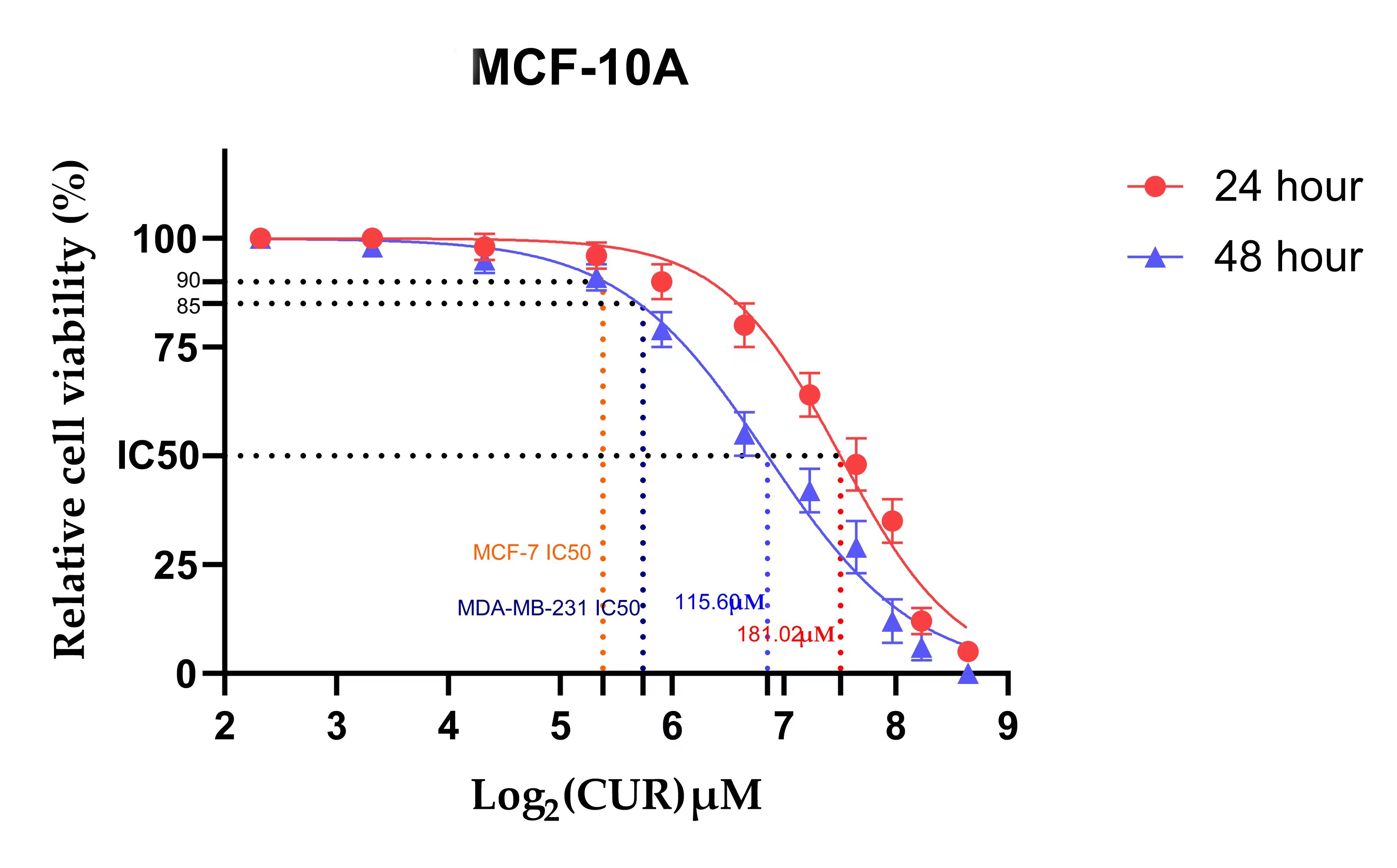

Supplement: Supplementary file 1 [file ijms-24-12479-s001.zip › figure s1.jpg]
